# EDTA aggregates induce SYPRO orange-based fluorescence in thermal shift assay

**DOI:** 10.1371/journal.pone.0177024

**Published:** 2017-05-04

**Authors:** Tobias Kroeger, Benedikt Frieg, Tao Zhang, Finn K. Hansen, Andreas Marmann, Peter Proksch, Luitgard Nagel-Steger, Georg Groth, Sander H. J. Smits, Holger Gohlke

**Affiliations:** 1 Institute for Pharmaceutical and Medicinal Chemistry, Heinrich Heine University Düsseldorf, Düsseldorf, Germany; 2 Institute for Physical Biology, Heinrich Heine University Düsseldorf, Düsseldorf, Germany; 3 Institute of Complex Systems, Structural Biochemistry (ICS-6), Forschungszentrum Jülich, Jülich, Germany; 4 Institute for Pharmaceutical Biology and Biotechnology, Heinrich Heine University Düsseldorf, Düsseldorf, Germany; 5 Institute for Biochemical Plant Physiology, Heinrich Heine University Düsseldorf, Düsseldorf, Germany; 6 Institute for Biochemistry, Heinrich Heine University Düsseldorf, Düsseldorf, Germany; Kermanshah University of Medical Sciences, ISLAMIC REPUBLIC OF IRAN

## Abstract

Ethylenediaminetetraacetic acid (EDTA) is widely used in the life sciences as chelating ligand of metal ions. However, formation of supramolecular EDTA aggregates at pH > 8 has been reported, which may lead to artifactual assay results. When applied as a buffer component at pH ≈ 10 in differential scanning fluorimetry (TSA) using SYPRO Orange as fluorescent dye, we observed a sharp change in fluorescence intensity about 20°C lower than expected for the investigated protein. We hypothesized that this change results from SYPRO Orange/EDTA interactions. TSA experiments in the presence of SYPRO Orange using solutions that contain EDTA-Na^+^ but no protein were performed. The TSA experiments provide evidence that suggests that at pH > 9, EDTA^4-^ interacts with SYPRO Orange in a temperature-dependent manner, leading to a fluorescence signal yielding a “denaturation temperature” of ~68°C. Titrating Ca^2+^ to SYPRO Orange and EDTA solutions quenched fluorescence. Ethylene glycol tetraacetic acid (EGTA) behaved similarly to EDTA. Analytical ultracentrifugation corroborated the formation of EDTA aggregates. Molecular dynamics simulations of free diffusion of EDTA-Na^+^ and SYPRO Orange of in total 27 μs suggested the first structural model of EDTA aggregates in which U-shaped EDTA^4-^ arrange in an inverse bilayer-like manner, exposing ethylene moieties to the solvent, with which SYPRO Orange interacts. We conclude that EDTA aggregates induce a SYPRO Orange-based fluorescence in TSA. These results make it relevant to ascertain that future TSA results are not influenced by interference between EDTA, or EDTA-related molecules, and the fluorescent dye.

## Introduction

Ethylenediaminetetraacetic acid (EDTA) and its parent molecule ethylene glycol tetraacetic acid (EGTA) are widely used in biology, biochemistry, pharmaceutical industry, and food technology because of their function as hexadentate chelating ligands of metal ions with a charge ≥ 2 [1]. However, interactions between EDTA and a protein, fibrinogen, leading to artifactual results, have been described in the context of fibrin polymerization [2, 3]. These interactions were related to the formation of supramolecular aggregates of EDTA at pH > 8 [1], which indicates an involvement of EDTA^3-^ or EDTA^4-^ (p*K*_a_ of the two tertiary amino groups in EDTA: 6.1 and 10.3 [4]). While it was also suggested that the aggregates may form supramolecular structures similar to lipid bilayers or micelles due to their strongly polarized nature at basic pH, no structural model at the atomistic level has been put forward [1].

**Fig 1:**
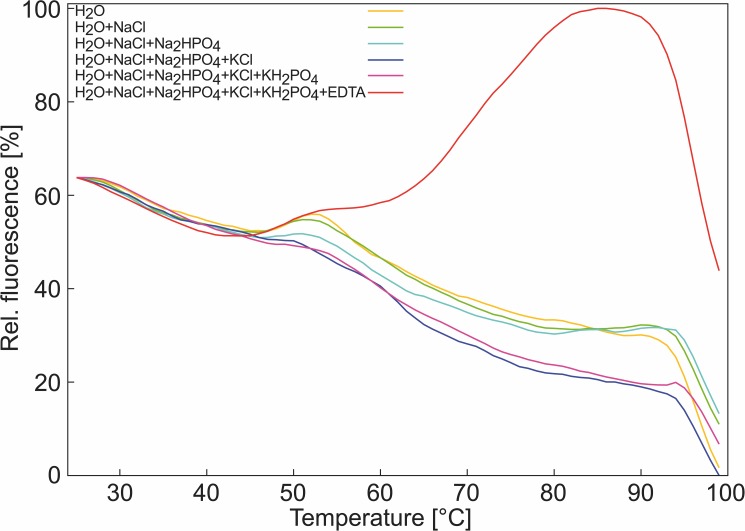
Thermofluor assay signal in the absence of protein. Fluorescence signal of SYPRO Orange in: H_2_O (yellow); H_2_O + 137 mM NaCl (green); H_2_O + 137 mM NaCl + 1.5 mM NaH_2_PO_4_ (blue); H_2_O + 137 mM NaCl + 1.5 mM NaH_2_PO_4_ + 2.7 mM KCl (dark blue); H_2_O + 137 mM NaCl + 1.5 mM NaH_2_PO_4_ + 2.7 mM KCl + 1.5 mM KH_2_PO_4_ (purple); H_2_O + 137 mM NaCl + 1.5 mM NaH_2_PO_4_ + 2.7 mM KCl + 1.5 mM KH_2_PO_4_ + 100 mM EDTA (red); the signals were normalized to the overall highest detected signal.

Recently, we intended to establish a differential scanning fluorimetry assay (DSF; also termed thermal shift assay, TSA) to measure the thermal denaturation temperature of a protein for which we needed to use EDTA to prevent precipitation. In TSA, the protein-containing solution is analyzed using real-time PCR machines after the addition of a fluorescent dye [5–7], in our case SYPRO Orange [8]. SYPRO Orange originated as a stain for protein bands in SDS gels, where it interacts with the SDS coat around the proteins in the gel [8]. In water, SYPRO Orange has a low quantum yield, resulting in a low fluorescence intensity at low temperatures in the TSA [9]. With increasing temperature, the protein unfolds, exposing hydrophobic residues to the solvent and SYPRO Orange, which results in an increase in fluorescence intensity [9]. In our assay, we obtained a satisfying signal-to-noise ratio only at pH ≈ 10. However, we failed to reproduce the melting temperature of the protein of ~86°C previously measured by CD spectroscopy [10]; instead, we observed a sharp change in fluorescence intensity at 68°C (Fig 1). We hypothesized that this change results from SYPRO Orange/EDTA interactions rather than from interactions of SYPRO Orange with the unfolded protein.

To test our hypothesis, we performed TSA experiments in the presence of SYPRO Orange using solutions that contain EDTA-Na^+^, or EGTA-Na^+^, but no protein, at varying concentrations, pH values, and in the presence or absence of Ca^2+^. Furthermore, we performed all-atom molecular dynamics (MD) simulations of free diffusion of EDTA-Na^+^ and SYPRO Orange under conditions closely resembling the TSA experiments. We provide evidence that suggests that at a practically relevant pH > 9 [11, 12], EDTA in its quadruple negatively charged state interacts with SYPRO Orange in a temperature-dependent manner, leading to a fluorescence signal similar to that when proteins unfold, with a “denaturation temperature” of ~68°C under the conditions chosen here. Furthermore, we suggest the first structural model of EDTA aggregates at the atomistic level and how these can interact with SYPRO Orange. Given the widespread use of EDTA in biology, pharmacy, and food technology, and TSA for investigating the thermal stability of proteins under varying conditions [13], our results make it relevant to ascertain that future TSA results are not influenced by interference between the chelator and the fluorescent dye.

## Materials and methods

### Materials

Ethylenediaminetetraacetic acid (EDTA) disodium salt dihydrate and SYPRO® Orange (5000x stock solution; 10 mM [14]) were from Sigma (Deisenhofen, Germany).

### Differential scanning fluorimetry

TSA was carried out with the real-time thermo-cycler qTOWER 2.0 (Analytik Jena AG, Germany) with the fluorescent dye SYPRO Orange (1:1000) in 96 well PCR plates. The fluorescence signal was initially measured at a temperature of 25°C, which was then increased to 100°C with a step size of 1°C/min. An interaction with a hydrophobic surface increases the quantum yield of the dye [15, 16]. Fluorescence changes in the wells of the plate were monitored simultaneously with a Channel photo multiplier. The wavelengths for excitation and emission were 490 nm and 580 nm, respectively. For each experiment, up to 12 different conditions were tested; see S1 Table for a summary of the composition of the samples. For statistical analysis, experiments were carried out with at least *n* = 8 samples per condition. Results are expressed as mean values ± standard error of the mean (SEM) and compared using a two-sided Student’s *t*-test. EC_50_ values were calculated with PRISM (GraphPad) using the log (inhibitor**)** *vs*. normalized response.

### Molecular dynamics simulations

In order to investigate SYPRO Orange/EDTA interactions at an atomistic level, we performed a set of MD simulations of SYPRO Orange in the presence of EDTA. Initial SYPRO Orange and EDTA 3D structures were generated using the Maestro 9.5 [17] program suite. SYPRO Orange molecules were generated according to the structural formula given in S1 Fig. As to EDTA, we prepared two variants that differ in terms of their protonation states. First, we prepared EDTA with three deprotonated and one protonated carboxyl groups (further referred as EDTA^3-^). Second, we prepared EDTA with four deprotonated carboxyl groups (further referred as EDTA^4-^). All structures were subjected to quantum mechanical geometry optimization, conducted with Gaussian 09 [18], using the HF/6-31G* basis set. The HF/6-31G* optimized structures were later used as initial structures for subsequent MD simulations.

Initially, we performed MD simulations on systems containing 20 EDTA^3-^ or EDTA^4-^ molecules, according to a pH < 9 or pH > 10, respectively, and two SYPRO Orange molecules. Therefore, the respective HF/6-31G* optimized structures were randomly placed using *PACKMOL* [19]. These structures were solvated by TIP3P water [20] using the *LEaP* program [21] of the AMBER 12 program suite [22], resulting in concentrations of 125 mM and 12 mM for EDTA and SYPRO Orange, respectively. To neutralize the systems, we added sodium counter ions (Na^+^), which are also part of the PBS buffer in our experiments, using *LEaP* [21]. Analogously, we prepared a set of additional systems for MD simulations, to investigate the influence of different experimental conditions on SYPRO Orange/EDTA interaction. Thus, we, first, prepared systems that contain only 16 EDTA^3-^ or EDTA^4-^ molecules and one SYPRO Orange, resulting in concentrations of 100 mM and 6 mM, which is in good agreement with our experimental procedure. Second, we prepared a system containing 20 EDTA^4-^ and two SYPRO Orange molecules, but leaving additional Na^+^ ions, to investigate the dependence of EDTA aggregation on the presence of Na^+^ ions. Here, we ensured neutrality of the system, by applying a uniform neutralizing plasma [23], present in particle mesh Ewald simulations with periodic boundaries in Amber 12 [22]. Third, to investigate whether SYPRO Orange/EDTA interactions are affected by EDTA complexation of calcium (Ca^2+^), we created a system, such that we randomly placed 20 octahedral EDTA^4-^—Ca^2+^ complexes and 2 SYPRO Orange molecules. Here, Na^+^ ions were added to neutralize the system. The octahedral EDTA^4-^—Ca^2+^ complexes were derived by short MD simulations of one EDTA^4-^ molecule and two Ca^2+^ ions in TIP3P water [20], beforehand.

As to both EDTA variants and SYPRO Orange, force field parameters were taken from the General AMBER Force Field GAFF [24]. Atomic partial charges were derived according to the restraint electrostatic potential fit (RESP) procedure [25]. Previous single point calculations were conducted with Gaussian 09 [18]. As to Na^+^ ions, parameters were taken from the AMBER force field ff12SB, which is distributed with the AMBER 12 suite of programs [22]. Additionally, to investigate the force field dependence of EDTA aggregation, we used ion parameters described by Joung and Cheatham [26], which have been improved with respect to the ions’ solution properties (the respective MD simulation is explicitly mentioned in the main text). As to Ca^2+^ ions, force field parameters were taken from Bradbrook *et al*. [27].

Subsequent minimization, thermalization, and production calculations were performed with the *pmemd*.*cuda* module [28] in Amber 12 [22]. We applied the MD protocol previously described here [29, 30]. In short, we performed three individual rounds of energy minimization with high, low, and no positional restraints applied to all solute atoms. In general, NVT production simulations were performed at 300.0 K (~ 27°C) for 1.0 μs. However, in order to investigate whether EDTA aggregation is also affected by increased temperatures, we performed two production simulations at 333.15 K (60°C) and 353.15 K (80°C), which are relevant for TSA experiments (the respective MD simulations are explicitly mentioned in the main text). The particle mesh Ewald method [31, 32] was applied to treat long-range electrostatic interactions. Conformations were stored to a trajectory file every 20 ps. All MD simulations described in this work accumulate to a total simulation time of 27 μs.

Analyses of the MD trajectories were performed using *cpptraj* [33] of AmberTools 14 [34]. The trajectories were analyzed with respect the EDTA solvent accessible surface area (SASA), as a measure for compactness of the EDTA aggregates. The EDTA shell around SYPRO Orange with a cutoff of 10 Å and the distance between the center of mass of the SYPRO Orange core region and the center of mass of the ethylene groups of the EDTA molecules were calculated, as a measure for interactions between SYPRO Orange and EDTA molecules. The radial distribution function *g*(*r*) between the carbon atoms of the ethylene group of EDTA molecules was calculated, ignoring intramolecular distances, as a measure of the probability of finding an ethylene group at a distance *r* away from a reference ethylene group.

## Results

### Interference of EDTA^4-^ and EGTA^4-^ with SYPRO Orange leads to an increase in fluorescence intensity in TSA

To scrutinize the origin of the unexpectedly low “denaturation temperature” observed during our initial TSA experiments on protein stability, we performed TSA experiments of water, PBS buffer (1.5 mM KH_2_PO_4_, 2.7 mM KCl, 10 mM Na_2_HPO_4_, 137 mM NaCl), and PBS buffer + EDTA-Na^+^ (25 mM) at pH 10 (adjusted with NaOH) in the presence of SYPRO Orange (1:1000 dilution according to previously performed experiments [35]); none of the solutions contained protein. The experiments reveal typical TSA curves [36, 37] of the relative change in fluorescence intensity in the presence of EDTA, with an inflection point at 68°C, whereas no increase in the fluorescence signal with increasing temperature was observed in the absence of EDTA (Fig 1). These results demonstrate an interference of EDTA and SYPRO Orange that leads to a temperature-dependent change in fluorescence intensity resembling that observed when a protein unfolds.

**Fig 2:**
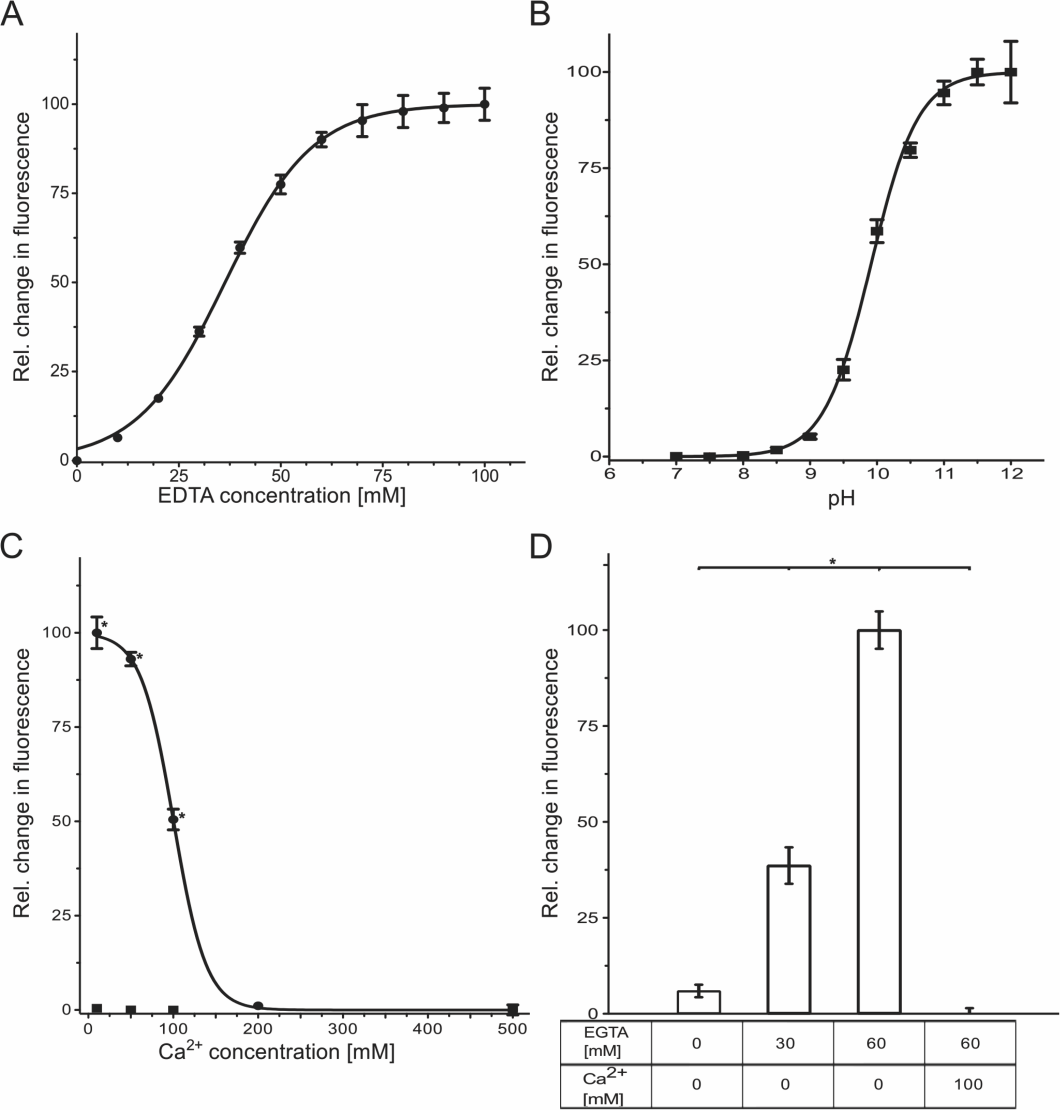
Influence of EDTA and EGTA on the SYPRO Orange-based fluorescence in a Thermofluor assay. (**A**) Dependence of the relative change in fluorescence intensity on the EDTA concentration (pH = 10); EC_50_ = 36.3 mM EDTA. (**B**) Dependence of the relative change in fluorescence intensity of the SYPRO Orange/EDTA system on the pH (100 mM EDTA); half-maximal change of fluorescence at pH = 9.9. (**C**) Dependence of the relative change in fluorescence intensity of the SYPRO Orange/EDTA system on the addition of Ca^2+^ (pH 10). Circles represent results for a sample in the presence of 100 mM EDTA, EC_50_ = 100 mM Ca^2+^; squares represent results for a sample in the absence of EDTA (negative control). (**D**) Dependence of the relative change in fluorescence intensity of the EGTA–SYPRO Orange system (pH = 10) on EGTA concentration and the absence or presence of Ca^2+^. (**A**)-(**D**): Values are normalized with respect to the minimal (0%) and maximal (100%) change detected. The error bars show the SEM. *: *p* < 0.0001.

In aqueous solutions containing SYPRO Orange (1:1000 dilution of the stock solution) and EDTA-Na^+^ at varying concentrations (pH 10), a concentration dependence of the relative change in fluorescence intensity with *EC*_50_ = 36.3 ± 0.6 mM EDTA was observed (Fig 2A; the composition of all samples shown in Fig 2 is listed in S1 Table). At these conditions, the *EC*_50_ value is equivalent to a molar ratio of SYPRO Orange:EDTA of 1:3.6. These findings demonstrate that more than one EDTA molecule must be present per SYPRO Orange for the temperature-dependent change in fluorescence intensity to occur. One can thus speculate that the change in fluorescence intensity is related to the formation of supramolecular aggregates of EDTA.

Depending on the pH, EDTA solutions contain molecular species with different charges: at 6.1 < pH < 10.3, EDTA^3-^ prevails, and at pH > 10.3 EDTA^4-^ [4].To investigate which EDTA species gives rise to the change in fluorescence intensity, we performed TSA experiments varying the pH between 7.0 and 12.0. For an aqueous solution of SYPRO Orange (1:1000 dilution) and EDTA (100 mM), an increase in the relative change in fluorescence intensity occurs at pH > 9, with a half-maximal relative change of fluorescence intensity at pH 10 (Fig 2B). At this pH, the concentration of the EDTA^4-^ species is ~27 mM in this solution. At pH 12, where the molar ratio of EDTA^4-^:EDTA^3-^ in aqueous solution is ~70:1, the relative fluorescence intensity is ~100%. These results demonstrate that it is the EDTA^4-^ species that is involved in the occurrence of fluorescence in the presence of SYPRO Orange.

EDTA^4-^ binds metal ions with a charge ≥ 2 usually through its four carboxylates and two amines, resulting in highly stable complexes with an octahedral geometry [38]. Titrating Ca^2+^ to aqueous solutions of SYPRO Orange (1:1000 dilution) and EDTA (100 mM) at pH 10 quenched the fluorescence with increasing Ca^2+^ concentrations, with *EC*_50_ = 100 ± 1.4 mM for the relative change in fluorescence intensity (Fig 2C). As a negative control, in the absence of EDTA, no influence of Ca^2+^ on the fluorescence intensity was observed (Fig 2C). This result demonstrates that EDTA^4-^, when complexed with Ca^2+^, cannot interfere with SYPRO Orange anymore. Furthermore, the result suggests that the interaction of EDTA^4-^ with SYPRO Orange is reversible, pointing to non-covalent SYPRO Orange/EDTA interactions.

In order to investigate if the observed interference with SYPRO Orange is EDTA^4—^specific, we performed TSA experiments with aqueous solutions of SYPRO Orange (1:1000 dilution) and EGTA (30–60 mM) at pH 10. EGTA is structurally closely related to EDTA in that a bis(2-aminoethoxy)ethylene moiety in the former replaces a 1,2-diaminoethylene moiety in the latter. As the largest p*K*_a_ of EGTA is 9.4, [39], the molar ratio of EGTA^4-^:EGTA^3-^ in aqueous solution of pH 10 is ~3.33:1. The EGTA^4-^ species binds Ca^2+^ similarly to the EDTA^4-^ species [40]. In contrast to using SYPRO Orange alone, a significant, concentration-dependent increase in the relative fluorescence intensity was observed in the presence of 30 and 60 mM EGTA (Fig 2D). At the latter conditions, the molar ratio of SYPRO Orange:EGTA is 1:6. The fluorescence was completely quenched when 100 mM Ca^2+^ were added (Fig 2D). Thus, EGTA, likely as an EGTA^4-^ species, also interferes with SYPRO Orange, probably in terms of supramolecular aggregates.

### Sedimentation velocity analysis

To analyze if EDTA forms aggregates that can enhance the fluorescence intensity of SYPRO Orange, we performed sedimentation velocity (SV) experiments by applying the fluorescence detection system, which permits excitation of SYPRO Orange at 488 nm. As shown in the collected concentration profiles, with purple lines being the early and red lines being the latest recordings (S2A Fig), the sedimentation boundary, which represents the distribution of fluorescent solutes in solution, moved gradually from the cell meniscus toward the cell bottom. This change in radial position of the sedimentation boundary reflects that fluorescent substances in solution depleted progressively from the meniscus to the bottom, clearly suggesting that these substances sedimented in the centrifugal force field over time. The c(*s*) distribution analysis (S2B Fig) showed that the average *s*_20,w_-value of the predominant species that sedimented in solution was 0.6 S. In addition, a small fraction of larger species at 2.15 S was detected. Finally, the weighted average fictional ratio *f/f*_*0*_ of the *c*(*s*) analysis was 2.2, indicating an extended, non-globular shape for the *s*-value species. In contrast to the sample containing EDTA, the fluorescence intensity in the sample with SYPRO Orange alone was too low to be accurately evaluated (data not shown). The SV analysis substantiated that EDTA formed supramolecular aggregates at high pH, which might lead to a significant fluorescence enhancement of SYPRO Orange.

### Structure elucidation of SYPRO Orange

Two structures of orange-colored fluorescent SYPRO^®^ stains have been described that differ in the length of the alkyl chains at the aniline nitrogen [41]. Since we could not obtain any information as to which one of the homologs is present in the commercial SYPRO Orange solution used here, we performed liquid chromatography-mass spectrometry and NMR spectroscopy (S3 and S4 Figs; see S1 Text for further details). The structure elucidation reveals two hexyl chains at the aniline nitrogen and a *trans* configuration of the double bond, resulting in the structural formula shown in S1 Fig.

### Structural model of EDTA supramolecular aggregates

Previously, the presence of supramolecular aggregates in basic solutions of ~4 mM EDTA-Na^+^ has been suggested, with a more evident effect observed when the EDTA^4-^ species is present [1]. However, no structural model of supramolecular aggregates of EDTA at the atomistic level has been put forward thus far. In order to generate such a structural model and to elucidate how SYPRO Orange can interfere with the aggregates, eventually leading to an increase in fluorescence intensity, we performed a series of all-atom MD simulations of free diffusion of EDTA-Na^+^ and SYPRO Orange in explicit solvent.

**Fig 3:**
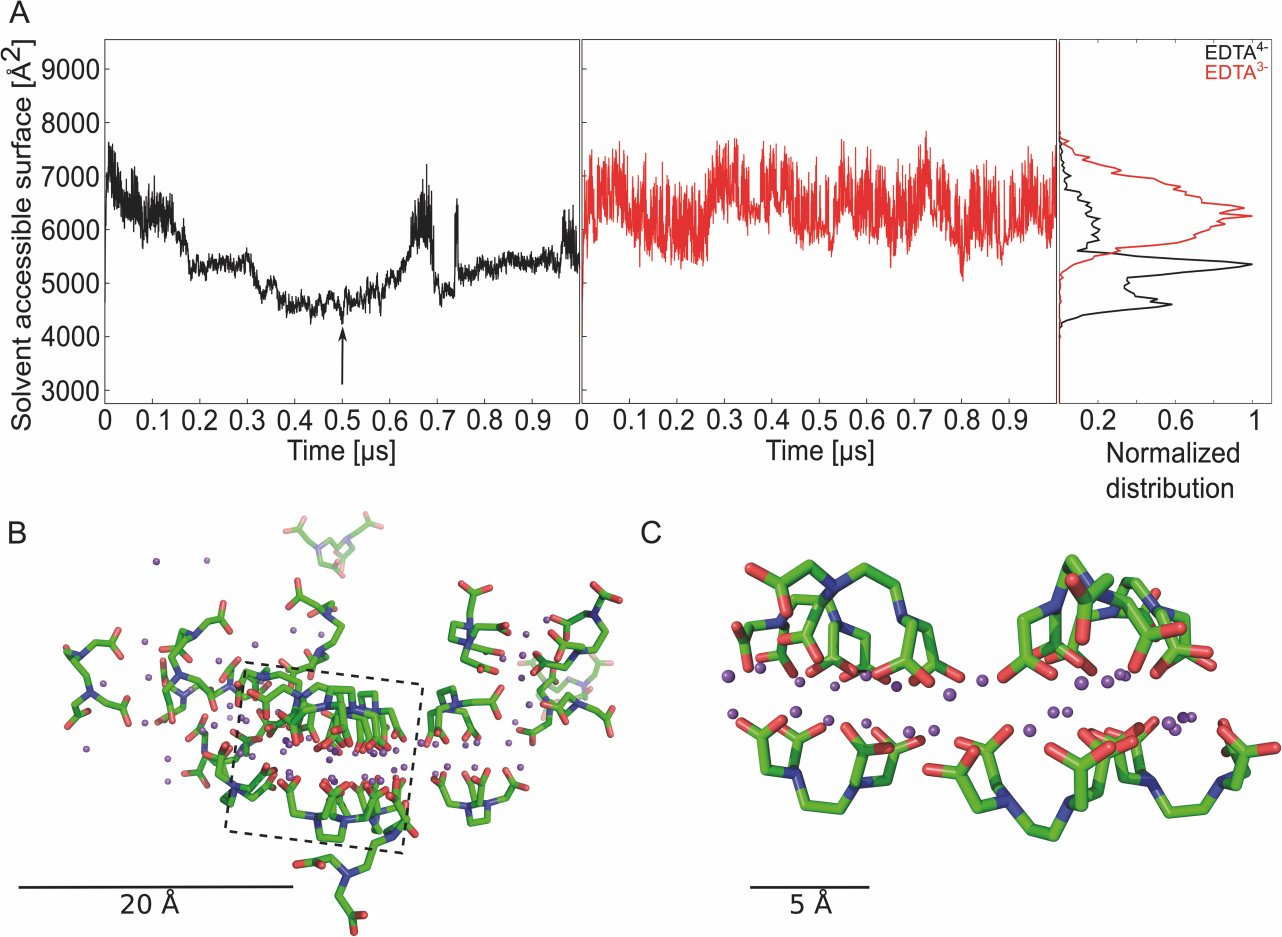
MD simulations to investigate EDTA aggregation and interaction with SYPRO Orange. (**A**) SASA and frequency distribution of the SASA over all 20 EDTA molecules in the simulation box; EDTA^4-^ (black), EDTA^3-^ (red), the arrow shows which snapshot was used to visualize the EDTA aggregate. (**B**) Snapshot of an MD trajectory showing an EDTA^4—^Na^+^ aggregate; the snapshot was taken at 500 ns (see arrow in panel A); EDTA^4-^: green: C atoms, blue: N atoms, red: O atoms; magenta: Na^+^ ions. Box: This part of the image was zoomed to get a better view on the bilayer within the aggregate. For clarity, some EDTA molecules in front of the bilayer were discarded. (**C**) Zoomed view on an EDTA^4—^Na^+^ aggregate showing a bilayer formation.

Initially, we performed MD simulations of 1 μs length on a system containing 20 EDTA^4-^ molecules, according to a pH >> 10, and two SYPRO Orange molecules in the simulation box (Fig 3), resulting in concentrations of 125 mM and 12 mM, respectively. As a control, MD simulations with the same number of molecules but now considering the EDTA^3-^ state were performed. In both cases, sodium counter ions were added, which are also part of the PBS buffer, to neutralize the systems. By visual inspection of the trajectories, we observed EDTA aggregates in the case of EDTA^4-^, but not for EDTA^3-^, which is in line with the observation of Müller *et al*. [1]. In order to quantify EDTA aggregation, we measured the SASA for all EDTA molecules, which gets smaller when EDTA aggregates (Fig 3A). As to EDTA^4-^, the SASA decreases from initially ~7000 Å^2^ by ~40% to ~4400 Å^2^ after 500 ns. Interestingly, the SASA increases after ~700 ns, which is related to a temporary collapse of the EDTA^4-^ aggregate showing the reversibility of its formation; the aggregate reconstitutes afterwards as shown again by a decrease in SASA. As to EDTA^3-^, the SASA fluctuates around the starting value of ~6300 Å^2^ and remains > 5300 Å^2^ throughout the MD simulations (Fig 3A). The frequency distributions of the SASA reveal two major populations in the case of EDTA^4-^ (peaks at ~5000 Å^2^ and ~4500 Å^2^) and one for EDTA^3-^ (peak at ~ 6300 Å^2^) (Fig 3A). These results indicate that the EDTA^4-^ species forms aggregates, but not the EDTA^3-^ species. Visual inspection revealed that the EDTA^4-^ aggregates are bilayer-like, with a layer of sodium ions separating two layers of EDTA^4-^ (Fig 3B). The negatively charged carboxylic functions of EDTA^4-^ encompass a cloud of positively charged sodium ions (Fig 3C), resulting in a bent conformation of the EDTA^4-^ molecules, that way exposing their ethylene moiety to the bilayer outside.

To investigate the force field dependence of the above described EDTA^4-^ aggregation, we again performed MD simulations of 1 μs length with 20 EDTA^4-^ molecules, two SYPRO Orange molecules, and 80 sodium ions, now using force field parameters for ions from the work of Joung and Cheatham [42]. Compared to the parameters of Åqvist [43] used above, those of Joung and Cheatham have been improved with respect to the ions’ hydration behavior [42]. Again, the SASA of the EDTA^4-^ molecules decreases from initially ~6000 Å^2^ by ~40% to ~3700 Å^2^ indicating EDTA^4-^ aggregation (S5 Fig). This result shows that the observed EDTA^4-^ aggregation occurs independently from specific ion parameters.

Next, we investigated if an EDTA concentration reduced by 20% (resulting in 16 EDTA^4-^ or EDTA^3-^ molecules in the simulation box equivalent to a concentration of 100 mM used in our experiments (S1 Table)) still leads to aggregation. During MD simulations of 1 μs length, the SASA of EDTA^4-^ molecules decreases from initially ~8000 Å^2^ by ~40% to ~4800 Å^2^ with a peak of the frequency distribution at 5400 Å^2^ (S6 Fig), whereas the SASA of EDTA^3-^ molecules remains > 5500 Å^2^ throughout the MD simulations, resulting in a distribution function that peaks at 6900 Å^2^ (S6 Fig). These results indicate that EDTA^4-^ forms aggregates under conditions that are similar to experiment, but not EDTA^3-^.

To investigate whether the observed EDTA aggregation depends on the presence of the sodium counter ions, we performed MD simulations of 1 μs length of 20 EDTA^4-^ molecules without counter ions. Instead, we applied a uniform neutralizing plasma to reach electroneutrality [23]. Contrary to the above observations and in line with expectations, the SASA of EDTA^4-^ molecules remains at ~9200 Å^2^ throughout the MD simulations (S7 Fig). This result shows that EDTA^4-^ aggregation does not occur in the absence of Na^+^ ions.

Next, we investigated whether the EDTA aggregation is affected by the complexation of Ca^2+^. Therefore, we created 20 EDTA^4—^Ca^2+^ complexes with an octahedral coordination of Ca^2+^ [38] in the simulation box and performed MD simulations of 1 μs length. In order to keep the system similar to our initial MD setup, 80 Na^+^ ions were added to the system. To reach electroneutrality, a uniform neutralizing plasma was applied [23]. In contrast to the above MD simulations, the initial SASA of the EDTA^4-^ molecules is only ~6100 Å^2^ due to the more compact shape of the EDTA^4—^Ca^2+^ complexes (S8A Fig). During the MD simulations, the SASA decreases to ~4500 Å^2^, resulting in a relative decrease of ~25%, only ~60% of that observed for EDTA^4-^ without Ca^2+^. Note that the ratio of EDTA^4-^:Ca^2+^ is 1:1 (125 mM each) at the simulation conditions; this ratio equals the ratio in our TSA experiments at *EC*_50_ = 100 mM Ca^2+^ in the presence of 100 mM EDTA (Fig 2C). Hence, only ~50% of the EDTA molecules are expected to form EDTA^4—^Ca^2+^ complexes in our MD simulations. Although the accurate description of calcium ions by classical MD simulations is complicated by strong electrostatic and polarization interactions with its surroundings due to its divalent nature [44], these data indicate that complex formation with Ca^2+^ can strongly reduce aggregate formation of EDTA^4-^, depending on the molar ratio of EDTA^4-^:Ca^2+^. In addition, visual inspection of the MD trajectory did not reveal a bilayer-like configuration of the EDTA^4-^ molecules but rather a fluffier configuration (S8B Fig).

To investigate whether EDTA aggregation is affected by increased temperatures, we performed MD simulations of 1 μs length of 20 EDTA^4-^ and EDTA^3-^ molecules at 333.15 K (60°C) and 353.15 K (80°C), 33.15 and 53.15 K higher than the above MD simulations. With respect to our TSA experiments, the former temperature is 8 K below the “denaturation temperature” of ~68°C (= 341.15 K) (Fig 1), whereas the latter temperature is 12 K above it. At 333.15 K, the SASA of EDTA^4-^ molecules decreases from initially ~6000 Å^2^ by ~50% to ~3100 Å^2^, whereas the SASA of EDTA^3-^ molecules fluctuates around ~5500 Å^2^ (S9A Fig). Very similar results are obtained for the MD simulations at 353.15 K (S9B Fig). The frequency distributions of SASA for EDTA^4-^ peak at ~3750 Å^2^ (333.15 K) and ~3200 Å^2^ (353.15 K), > 1600 Å^2^ lower than in the case of the MD simulations at 300 K (S9A and S9B Fig). In line with these data, the EDTA^4—^Na^+^ aggregates display a bilayer-like configuration (S9D Fig) that appears more compact than at 300 K (Fig 3B). Although these results may be influenced in that the TIP3P water model used in the MD simulations displays difficulties reproducing experimental values for water at higher temperatures [45, 46], our results indicate that increasing temperature fosters EDTA^4-^ aggregation, but does not influence the solution behavior of EDTA^3-^.

In order to gain further insight into the structure of the EDTA aggregates under different conditions we calculated the radial distribution function (*g*(*r*)) of carbon atoms of the EDTA ethylene moiety, ignoring intramolecular distances. *g*(*r*) describes how density varies as a function of the distance *r* from a reference particle. In the case of EDTA^4-^ in the presence of Na^+^ at 300 K, *g*(*r*) shows two prominent peaks at ~ 6 Å and ~11 Å (S10 Fig). The peak at short distance relates to carbon atoms of ethylene groups of two EDTA molecules next to each other on one side of the bilayer, whereas the peak at large distance relates to carbon atoms of ethylene groups of two EDTA molecules on opposite sides of the bilayer (Fig 3B and 3C); *g*(*r*) thus reveals that most of the EDTA^4-^ molecules are found in a bilayer-like configuration in the presence of Na^+^. In the absence of Na^+^, a broad peak is found between 15 and 20 Å, indicating the absence of short-range order, in agreement with our findings above (S10 Fig). In contrast, EDTA^4-^ in the presence of Na^+^ at 353.15 K shows more pronounced peaks at ~ 6 Å and ~11 Å than at 300 K (S10 Fig), again indicating that increasing temperature fosters EDTA^4-^ aggregation in a bilayer-like configuration. Notably, the presence of Ca^2+^ changes the short-range order of EDTA^4-^ in that only a peak at 11 Å is visible (S10 Fig). The missing peak at 6 Å indicates that the ethylene moieties of EDTA^4-^ do not come close to each other, in line with the fluffier configuration exemplarily shown in S8B Fig.

Overall, our results provide for the first time insights at the atomistic level into the aggregation behavior of EDTA^4—^Na^+^ and suggest a structural model of such aggregates.

### Structural model of the SYPRO Orange/EDTA interaction

**Fig 4:**
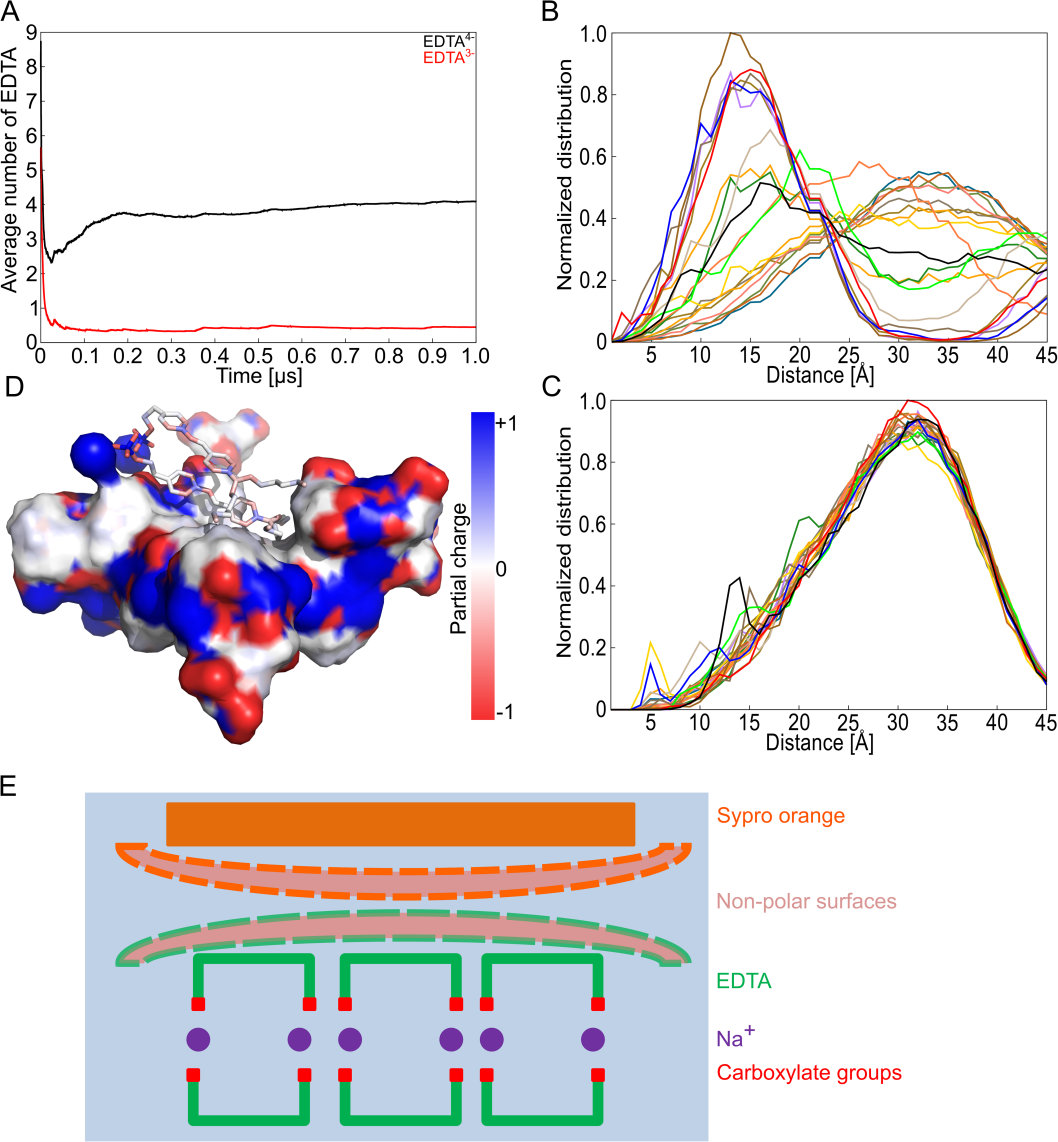
Structural model from MD simulations of EDTA aggregates and SYPRO Orange forming a complex with the hydrophobic region of EDTA. (**A**) Average number of EDTA molecules within 5 Å of the core region of SYPRO Orange for EDTA^4-^ (black) and EDTA^3-^ (red). The core region is defined as the two aromatic rings with the linker (see S1 Fig). (**B**) Frequency distribution of the distance of the center of the ethylene group of every EDTA^4-^ molecule to the center of mass of the core region (see panel A) of SYPRO Orange. (**C**) Frequency distribution of the distance of the center of the ethylene group of every EDTA^3-^ molecule to the center of mass of the core region (see panel A) of SYPRO Orange. Individual distance distributions are depicted in S10 Fig. The force field ff12SB was applied in both MD simulations. (**D**) Snapshot of an MD trajectory showing the EDTA^4—^Na^+^ aggregate from Fig 3B (surface representation) to which two SYPRO Orange molecules bind non-covalently. The coloring of the surface is according to the local partial atomic charge. (**E**) Schematic figure showing the association of SYPRO Orange with an EDTA^4—^Na^+^ aggregate as derived from panel D. The two non-polar surfaces of the aggregate and SYPRO Orange are highlighted by dashed borders.

In order to quantify interactions between EDTA and SYPRO Orange, we computed distance distributions between the center of mass of the ethylene groups of EDTA^4-^ or EDTA^3-^ molecules and the center of mass of the SYPRO Orange “core region” (for a definition of the “core region”, see S1 Fig) and used those for determining the number of EDTA^4-^ or EDTA^3-^ molecules around SYPRO Orange (termed “EDTA shell” hereafter). For this, the initial setup of the MD simulations with 20 EDTA^4-^ (EDTA^3-^) molecules, two SYPRO Orange molecules, and 80 (60) Na^+^ at 300 K was used (Fig 3). EDTA molecules were considered to belong to a shell around SYPRO Orange if the distance between the center of mass of the SYPRO Orange core region and the center of mass of the EDTA ethylene group was ≤ 10 Å. After 200 ns, a shell of ~4 EDTA^4-^ molecules is established around SYPRO Orange (Fig 4A). This ratio of SYPRO Orange:EDTA of 1:4 agrees very well with the ratio of SYPRO Orange:EDTA of 1:3.6 at the *EC*_50_ in our TSA experiment. In contrast, for EDTA^3-^, no shell is formed. In more detail, the distance distributions revealed two groups of EDTA^4-^ molecules with respect to their proximity to SYPRO Orange: The first group containing 55% of the molecules displays a peak in the distribution at ~15 Å; the second group (45%) displays one at ~35 Å (Fig 4B). In contrast, all EDTA^3-^ molecules displayed a peak in the distance distribution with respect to SYPRO Orange at ~33 Å (Fig 4C). Frequency distributions of the respective distances for each EDTA molecule are shown in S11 Fig. In the absence of any interactions between SYPRO Orange and EDTA, the average distance between the center of mass of the SYPRO Orange core region and the center of mass of the EDTA ethylene group is expected to be ~ 28 Å, equivalent to half of the size of the simulation box. These results indicate that EDTA^4-^ and SYPRO Orange interact, in contrast to EDTA^3-^ and SYPRO Orange. As SYPRO Orange is neutral at pH > 10, making different electrostatic interactions between the EDTA species and SYPRO Orange implausible, the differential behavior must be related to EDTA^4-^ forming aggregates.

An example for how SYPRO Orange interacts with an EDTA^4-^ aggregate is depicted in Fig 4D. Here, the surface of the aggregate and SYPRO Orange were colored according to the local partial atomic charges. The figure reveals that SYPRO Orange binds with its core region to the non-polar region of the aggregate formed by the ethylene moieties of EDTA^4-^; these non-polar interactions are expanded by the propyl and hexyl chains of the dye. A very similar binding mode is revealed by the exemplary aggregate configuration from the MD simulations at 353.15 K (S9E Fig).

Overall, our results provide evidence that suggest that SYPRO Orange interacts with EDTA^4-^ aggregates in a bilayer-like configuration via non-polar interactions.

## Discussion

In this study, we validated the hypothesis that the sharp change in fluorescence intensity observed in TSA experiments containing SYPRO Orange and EDTA at basic pH, but no protein, results from SYPRO Orange/EDTA interactions. To do so, we applied a combined experimental and computational approach using TSA and MD simulations. Our data provides evidence that suggests that at pH > 9, EDTA in its quadruple negatively charged state interacts with SYPRO Orange in a temperature-dependent manner, leading to a fluorescence signal similar to that when proteins unfold, with a “denaturation temperature” of ~68°C under the conditions chosen here. Furthermore, we suggest the first structural model of EDTA aggregates at the atomistic level and how these can interact with SYPRO Orange.

TSA experiments performed at least in octuplicates unequivocally demonstrated that typical TSA curves [36, 37] of the relative change in fluorescence intensity of SYPRO Orange occurred for water and PBS buffer solutions once EDTA at basic pH, but no protein, was present (Fig 1). The effect is EDTA concentration-dependent (Fig 2A), and the *EC*_50_ = 36.3 mM found is within the range of practically applied EDTA concentrations [47–49]. The effect is also pH-dependent (Fig 2B), and the half-maximal relative change of fluorescence intensity found at pH 10 relates to a concentration of the EDTA^4-^ species of ~27 mM in this solution, close to the *EC*_50_ value found above for the EDTA concentration dependence. A pH value of that magnitude is experimentally relevant when investigating proteins that have pH optima up to pH ~12 [11, 12]. The maximal relative fluorescence intensity was measured above pH 12. These results clearly identified the EDTA^4-^ species to be involved in the occurrence of fluorescence in the presence of SYPRO Orange. Titrating Ca^2+^ to aqueous SYPRO Orange and EDTA solutions quenched the fluorescence with increasing Ca^2+^ concentrations (Fig 2C), which may be due to either masking part of the EDTA charge, a change in the EDTA conformation because of the formation of octahedral EDTA^4—^Ca^2+^ complexes [38], and/or the dissociation of supramolecular aggregates of EDTA. The formation of such supramolecular aggregates has been demonstrated in previous work [1], and our finding of an SYPRO Orange:EDTA ratio of 1:3.6 at *EC*_50_ = 36.3 mM EDTA (Fig 2A) supports this result. We applied analytical ultracentrifugation (AUC) to corroborate the formation of such supramolecular aggregates; AUC is a widely used technique for the quantitative analysis of biomacromolecules in solution [50]. As one of the commonly used methods in AUC, SV analysis provides first-principle and hydrodynamic information on the shape and size of target molecules. The fluorescence detection system enables the evaluation of the sedimentation of fluorescent materials at a very low concentration [51]. In the SV analysis, we successfully detected the movement of the sedimentation boundary at 60,000 rpm over time, demonstrating that materials in solution indeed formed aggregates. Given a frictional ratio *f/f*_*0*_ of 2.2, we concluded that the aggregates should adopt certain types of structures other than a globular shape. The second species at 2.15 S correspond to some larger aggregates. Assuming a 1:5 stoichiometry for SYPRO Orange:EDTA, a molar mass for the 0.6 S species of about 2.5 kDa and for the 2.15 S species of about 18 kDa can be derived. The Ca^2+^ titration experiments furthermore suggested that the interaction of EDTA^4-^ with SYPRO Orange is reversible. Finally, very similar results obtained for SYPRO Orange and EGTA, in the absence of protein, with respect to the EGTA concentration dependence, pH, and Ca^2+^ influence (Fig 2D) suggest that changes in fluorescence intensity in TSA experiments with SYPRO Orange at basic pH might occur also for other members of the EGTA family of molecules.

While our TSA experiments demonstrated that EDTA^4-^ interference with SYPRO Orange can lead to an increase in fluorescence intensity in TSA even in the absence of a protein, the molecular origin of this effect remained elusive, not least because no structural model of supramolecular aggregates of EDTA at the atomistic level has been put forward [1]. We aimed at providing such a structural model as well as one of SYPRO Orange/EDTA interactions by all-atom MD simulations of free diffusion of EDTA-Na^+^ and SYPRO Orange of a total simulation time of 27 μs. MD simulations of free ligand diffusion have been applied successfully recently to elucidate binding modes, equilibria, and kinetics of protein-ligand [52–57] and host-guest systems [58, 59]. However, MD simulations of free molecule diffusion to study aggregation behavior face challenges related to force field accuracy [60, 61] and events that exceed the simulated timeframe [62, 63].

We addressed these challenges at four levels. First, we applied state-of-the-art parameterizations for the molecular models of EDTA and water in terms of the ff12SB force field from the AMBER 12 suite of programs [22] and the TIP3P water model [20]. The influence of the force field of Na+ ions on the aggregation behavior was tested with respect to parameterizations of Åqvist [43] as well as Joung and Cheatham [42], which differ in the ions’ hydration properties. However, the observed EDTA^4-^ aggregation was quantitatively similar in both cases (S5 Fig), indicating that the EDTA^4-^ aggregation occurs independently from specific ion parameters. Second, we performed MD simulations of 1 μs length each, which allowed to observe the temporary collapse and reformation of the EDTA^4-^ aggregate (Fig 3) and yielded almost constant SASA values in the last quarter of each MD simulation. Third, we aimed at validating our simulation setup by semi-quantitative comparisons of EDTA^4-^ aggregation in the presence and absence of Ca^2+^ (S8 Fig) and at different temperatures (S9 Fig) with respect to experiment. As to the former, the MD simulations revealed a decrease in the aggregation formation of 40% in the presence of a Ca^2+^ concentration equivalent to that in TSA experiments that led to only 50% of the relative change in fluorescence intensity. As to the latter, the MD simulations indicate that increasing temperature fosters EDTA^4-^ aggregation and that this effect is higher above the experimental “denaturation temperature” of SYPRO Orange/EDTA solutions than below. These results correspond to a higher fluorescence intensity found in TSA above the experimental “denaturation temperature” than below. Fourth, in our analyses, we focused on relative changes in molecular properties rather than absolute ones in order to exploit effects of cancellation of errors with respect to the used force fields. As such, we compared EDTA^4-^ aggregation properties to those of EDTA^3-^ (Figs 3 and 4; S6 and S9 Figs) and analyzed EDTA^4-^ aggregation properties at different concentrations (Fig 3; S6 Fig) and in the presence or absence of Na^+^ (Fig 3; S7 Fig). In this context, the MD simulations indicated that the EDTA^4-^ species forms aggregates, but not the EDTA^3-^ species, and that EDTA^4-^ aggregation occurs at concentrations similar to experiment but not in the absence of Na^+^ ions.

In the structural model of EDTA aggregates suggested by the MD simulations, U-shaped EDTA^4-^ molecules arrange such that they encompass Na^+^ ions between their carboxylate groups and expose their ethylene moieties to solvent (Fig 3B), that way forming an inverse bilayer. Such inverse bilayer structures have been observed for salts of amphiphilic anions in the solid state [64]. In our case, we attribute their occurrence to two points: First, the exposed ethylene moiety only forms a small hydrophobic surface and is flanked by two polar tertiary amine groups, resulting in a sufficient solubility in water, as inferred from the related compound *N*,*N*,*N*',*N*'-tetramethylethylendiamine, which is soluble in water and shows a log K_OW_ = 0.3 [65]. Second, in accordance with the “law of matching water affinities” [66], the small cation Na^+^ forms contact ion pairs with carboxylate groups particularly well, that way exhibiting specific binding properties in addition to nonspecific charge screening [67–70]. As a result, Na^+^ stabilizes x-poly-L-glutamate with ionized side chains in a helical conformation [68]. Furthermore, Na^+^, by way of bridging carboxylates at strongly conserved positions, stabilizes micelles [71] and superlattices [72] of amphiphilic carboxylates. There, RCOO^-^ … Na^+^ … ^-^OOCR ion triplets were found to be particularly important for aggregation [72]. As indicated by Fig 3B, we also find contact ion triplets in our EDTA^4-^ aggregate. Notably, the formation of contact ion triplets paradoxically reduces electrostatic repulsion when more carboxylic groups are deprotonated because the strong ionic bridging interactions replace weaker hydrogen bonds involving non-ionized carboxylic groups [72].

The structural model of the EDTA aggregate allows one to reconcile why it is formed by the EDTA^4-^ species but not the EDTA^3-^ species: Positive charges at the amine groups in EDTA^3-^ may lead to repulsive forces between neighboring EDTA molecules that destabilize the aggregate. The structural model is in line with suggestions from TSA experiments and MD simulations that aggregation is fostered by increasing temperature: Both the facts that the hydration free energy of carboxylate side chains is adversely affected by increasing temperature [73], and that the formation of contact ions pairs involving Na^+^ becomes more favorable with increasing temperature [74] can lead to stronger bridging interactions by contact ion triplets. Finally, although the size of the EDTA aggregate formed during the MD simulations may be limited by the number of EDTA molecules available in the simulation box, we note that its longest dimension is ~38 Å (Fig 3B) and ~31 Å (S9D Fig) and, hence, in fair agreement with a radius of 20–40 Å found for EDTA aggregates by cryo-electron microscopy [1]. The observation from MD simulations that SYPRO Orange binds with its mostly hydrophobic fluorophore moiety to the non-polar region of the EDTA aggregate formed by the ethylene moieties of EDTA^4-^ (Figs 3B and 4D; S9D and S9E Fig) may provide an explanation for the increase in fluorescence intensity observed in TSA due to a solvatochromic effect.[15, 16] The SYPRO Orange/EDTA interaction is reminiscent of the SYPRO Orange/SDS interaction suggested to lead to a fluorescence signal when staining protein bands in SDS gels [8]. Furthermore, increasing temperature is suggested to foster EDTA^4-^ aggregation, which should allow for improved SYPRO Orange/EDTA interactions, as seen by the increased number of EDTA^4-^ molecules within the shell around SYPRO Orange (S9C Fig); these results can explain the increase of fluorescence intensity observed with increasing temperature, resulting in a “denaturation temperature” of 68°C in our TSA experiments. In the presence of Ca^2+^ ions, the fluorescence signal is quenched (Fig 2C and 2D). This experimental finding agrees with our observation from MD simulations that EDTA^4-^ in that case does not form a bilayer-like configuration (S8B and S10 Figs), which results in a markedly smaller number of EDTA^4-^ molecules forming a shell around SYPRO Orange than in the absence of Ca^2+^ (S8C Fig) and, hence, reduced hydrophobic interactions between EDTA^4-^ and SYPRO Orange, leading to a reduced solvatochromic effect.

In summary, we provide evidence that suggests that EDTA^4-^ interacts with SYPRO Orange in a temperature-dependent manner, leading to a fluorescence signal in TSA similar to that when proteins unfold. Considering that EDTA is widely used in biology, pharmacy, and food technology, as is TSA for investigating the thermal stability of proteins under varying conditions, we hardly need to emphasize that our results make it relevant to ascertain that future TSA results are not influenced by interference between EDTA, or likely other members of the EGTA family, and the fluorescent dye.

## Supporting information

S1 Fig 2D structure of SYPRO Orange. Starting from the published structures (303/304), the length of the alkyl chains and the configuration at the double bond were assessed by mass spectrometry and NMR, respectively (S3 and S4 Figs).
(TIF)

S2 Fig SV analysis on 40 mM EDTA in the presence of 136 mM Na^+^ and 0.4% SYPRO Orange at pH 11. (A) Original sedimentation profile and *c*(*s*) fitting output of the sample. Real time data acquired by the detector were shown in colored dots, while the deconvolution results were displayed in colored curves. Fitting residuals were shown at the bottom of the graph. (B) The sedimentation coefficient distribution of the sample determined by *c*(*s*) model. Data were normalized according to the area under the curve and expressed as *s*_20,w_-values.
(TIF)

S3 Fig Mass spectra of SYPRO Orange. (A) Results for the pseudo molecular ion obtained by positive ionization with a proton; 487.3 Da. (B) Results with negative ionization with formate; 531.1 Da. The calculated mass for the investigated SYPRO Orange ion is 486 Da, in agreement with the structural formula shown in S1 Fig.
(TIF)

S4 Fig NMR spectroscopy to determine the configuration of the double bond in SYPRO Orange. (A) Full proton (^1^H) NMR spectrum of SYPRO Orange in DMSO-*d*_6_. (B) Zoom into the SYPRO Orange-specific part of the spectrum in panel A (between 7.0 and 8.0 ppm); the coupling constant of the protons at the double bond is ^3^*J* = 16 Hz, revealing a *trans* configuration of that bond.
(TIF)

S5 Fig Dependence of EDTA aggregation on the force field parameters for metal ions. Solvent accessible surface area (SASA) and frequency distribution of the SASA over all 20 EDTA^4-^ molecules in the simulation box (solid line). Here parameters for metal ions from Joung *et al*. were applied; all other simulation conditions were as in Fig 1. The dashed line in the frequency distribution has been added for comparison (see Fig 3A) and shows the results obtained with the parameters for metal ions from the ions94 library of Amber12.
(TIF)

S6 Fig Dependence of EDTA aggregation on the EDTA concentration. SASA and frequency distribution of the SASA over all 16 EDTA molecules in the simulation box; EDTA^4-^ (black), EDTA^3-^ (red). The dashed line in the frequency distribution has been added for comparison (see Fig 3A) and shows the results obtained with the originally applied concentration.
(TIF)

S7 Fig EDTA aggregation does not occur in the absence of Na^+^ ions. (A) SASA and frequency distribution of the SASA over all 20 EDTA^4-^ molecules in the simulation box in the absence of Na^+^ counter ions. The dashed lines in the frequency distribution has been added for comparison (see Fig 3A) and shows the results obtained in the presence of the counter ions.
(TIF)

S8 Fig EDTA aggregation in the presence of Ca^2+^. (A) SASA and frequency distribution of the SASA over all 20 EDTA^4-^ molecules in the simulation box. The dashed line in the frequency distribution has been added for comparison (see Fig 3A) and shows the results obtained without calcium ions but in the presence of Na^+^. (B) Snapshot of an MD trajectory showing an EDTA^4-^/Ca^2+^–Na^+^ aggregate; the snapshot was taken at 500 ns; EDTA^4-^: green: C atoms, blue: N atoms, red: O atoms; magenta: Na^+^ ions; orange: Ca^2+^ ions. (C) Average number of EDTA molecules within 5 Å of the core region of SYPRO Orange for EDTA^4-^ in the presence of Na^+^ (black) and in the presence of Ca^2+^ and Na^+^ (blue). The core region is defined as the two aromatic rings with the linker (see S1 Fig).
(TIF)

S9 Fig Temperature dependence of EDTA aggregation. (A) SASA and frequency distribution of the SASA over all 20 EDTA molecules in the simulation box at 333.15 K; EDTA^4-^ (black), EDTA^3-^ (red). (B) SASA and frequency distribution of the SASA over all 20 EDTA molecules in the simulation box at 353.15 K; EDTA^4-^ (black), EDTA^3-^ (red). The dashed lines in the frequency distribution have been added for comparison (see Fig 3A) and show the results obtained at 300 K. (C) Average number of EDTA molecules within 5 Å of the core region of SYPRO Orange for EDTA^4-^ (black) and EDTA^3-^ (red) at 353.15 K. The core region is defined as the two aromatic rings with the linker (see S1 Fig). (D) Snapshot of an MD trajectory showing an EDTA^4-^ –Na^+^ aggregate; the snapshot was taken from the MD simulations performed at 353.15 K at 500 ns; EDTA^4-^: green: C atoms, blue: N atoms, red: O atoms; magenta: Na^+^ ions. (E) Snapshot of an MD trajectory showing the EDTA^4-^ –Na^+^ aggregate from panel C (surface representation) to which two SYPRO Orange molecules non-covalently bind. The coloring of the surface is according to the local partial atomic charge.
(TIF)

S10 Fig Analysis of the distribution of EDTA molecules under various conditions. Radial distribution function of carbon atoms of the EDTA^4-^ ethylene moiety, ignoring intramolecular distances, at 300 K in the presence of Na^+^ (black), in the presence of Ca^2+^ and Na^+^ (blue),or in the absence of counterions (brown), and at 353.15 K in the presence of Na^+^ (green).
(TIF)

S11 Fig Proximity of EDTA and SYPRO Orange molecules. Frequency distributions of distances between the center of ethylene groups of each EDTA^4-^ (A) or EDTA^3-^ (B) molecule and the core region (see S1 Fig) of the nearest SYPRO Orange molecule.
(TIF)

S1 Table Composition of samples for Thermofluor assay. (PDF)

S1 Text Supporting methods. (PDF)
