# Supplementary figures and images for: EDTA aggregates induce SYPRO orange-based fluorescence in thermal shift assay
Source: PLoS One. 2017 May 4;12(5):e0177024. doi: 10.1371/journal.pone.0177024 (PMC5417642; doi:10.1371/journal.pone.0177024)

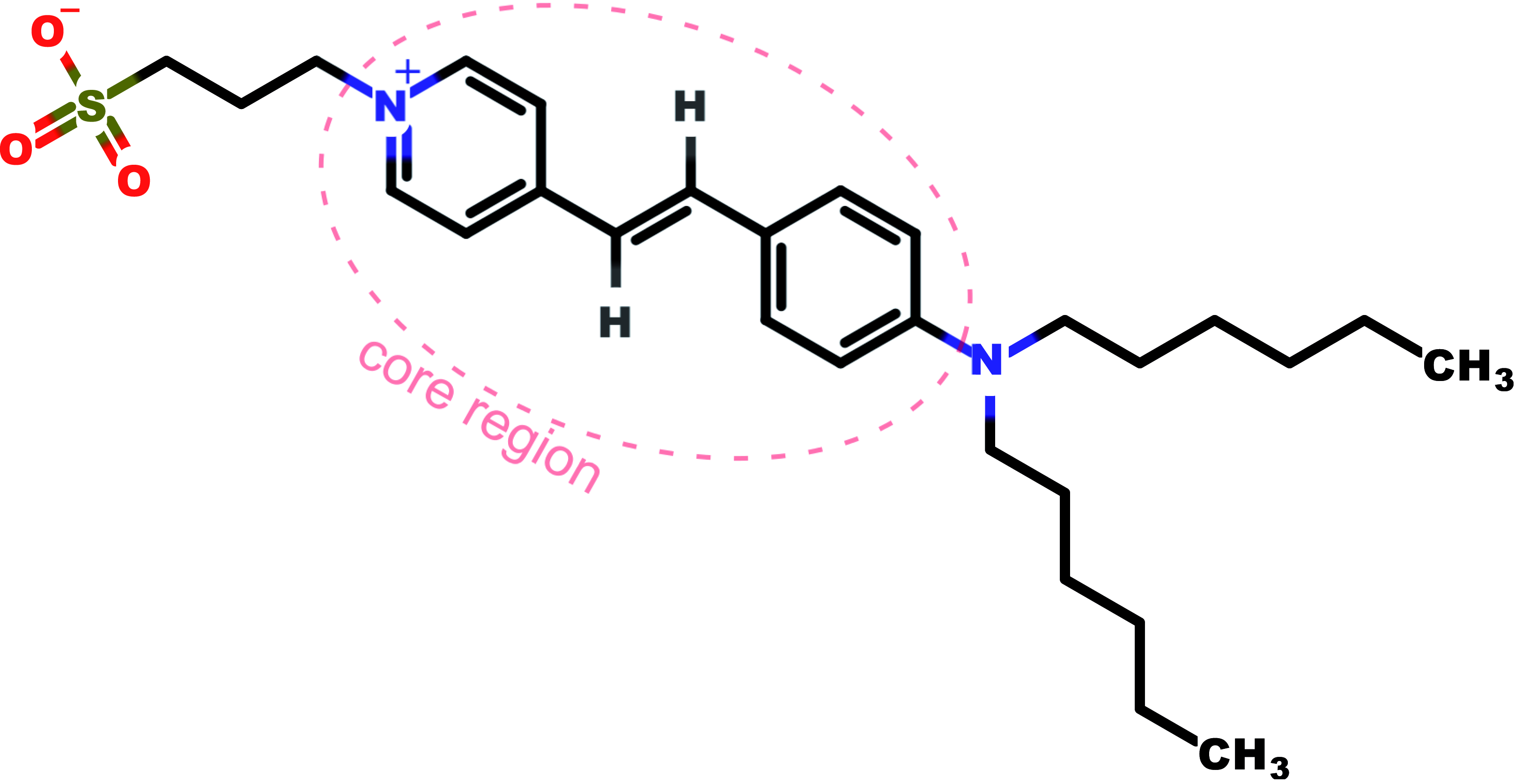

Supplement: S1 Fig — Starting from the published structures (303/304), the length of the alkyl chains and the configuration at the double bond were assessed by mass spectrometry and NMR, respectively (S3 and S4 Figs). (TIF) [file pone.0177024.s001.tif]

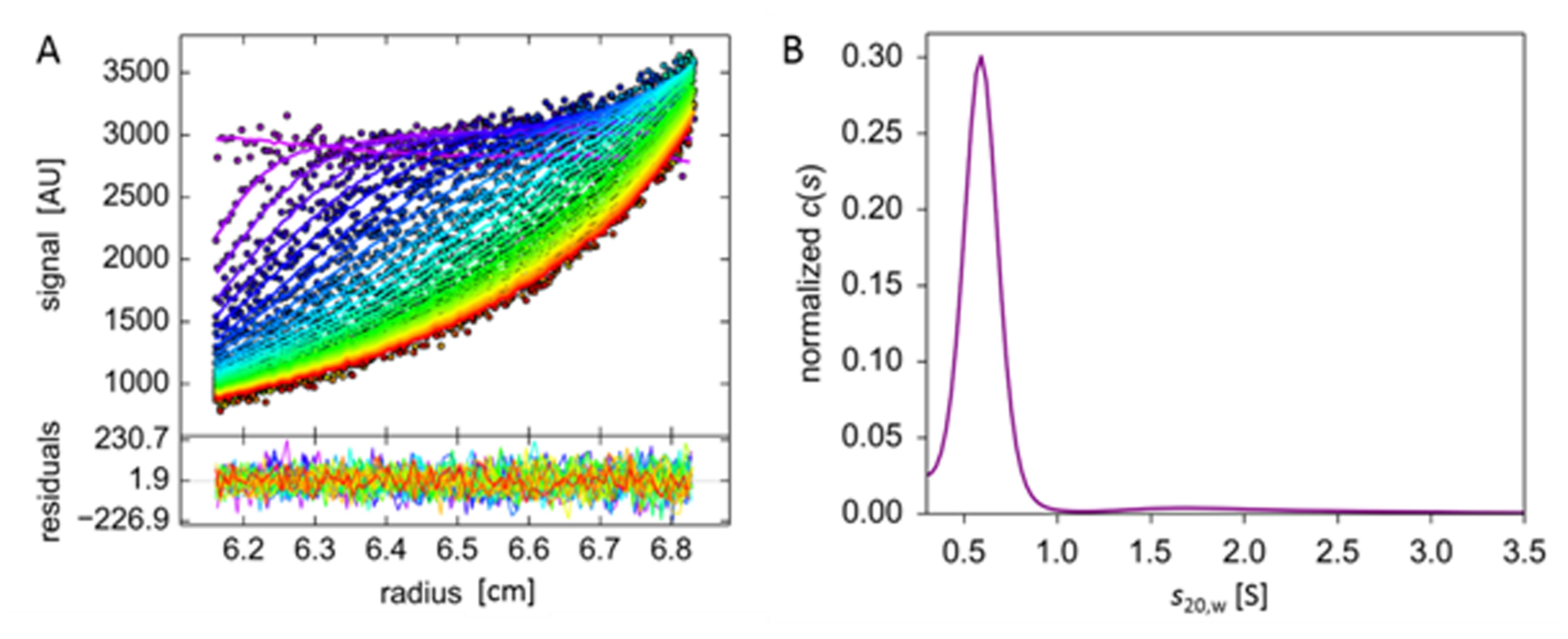

Supplement: S2 Fig — (A) Original sedimentation profile and c(s) fitting output of the sample. Real time data acquired by the detector were shown in colored dots, while the deconvolution results were displayed in colored curves. Fitting residuals were shown at the bottom of the graph. (B) The sedimentation coefficient distribution of the sample determined by c(s) model. Data were normalized according to the area under the curve and expressed as s20,w-values. (TIF) [file pone.0177024.s002.tif]

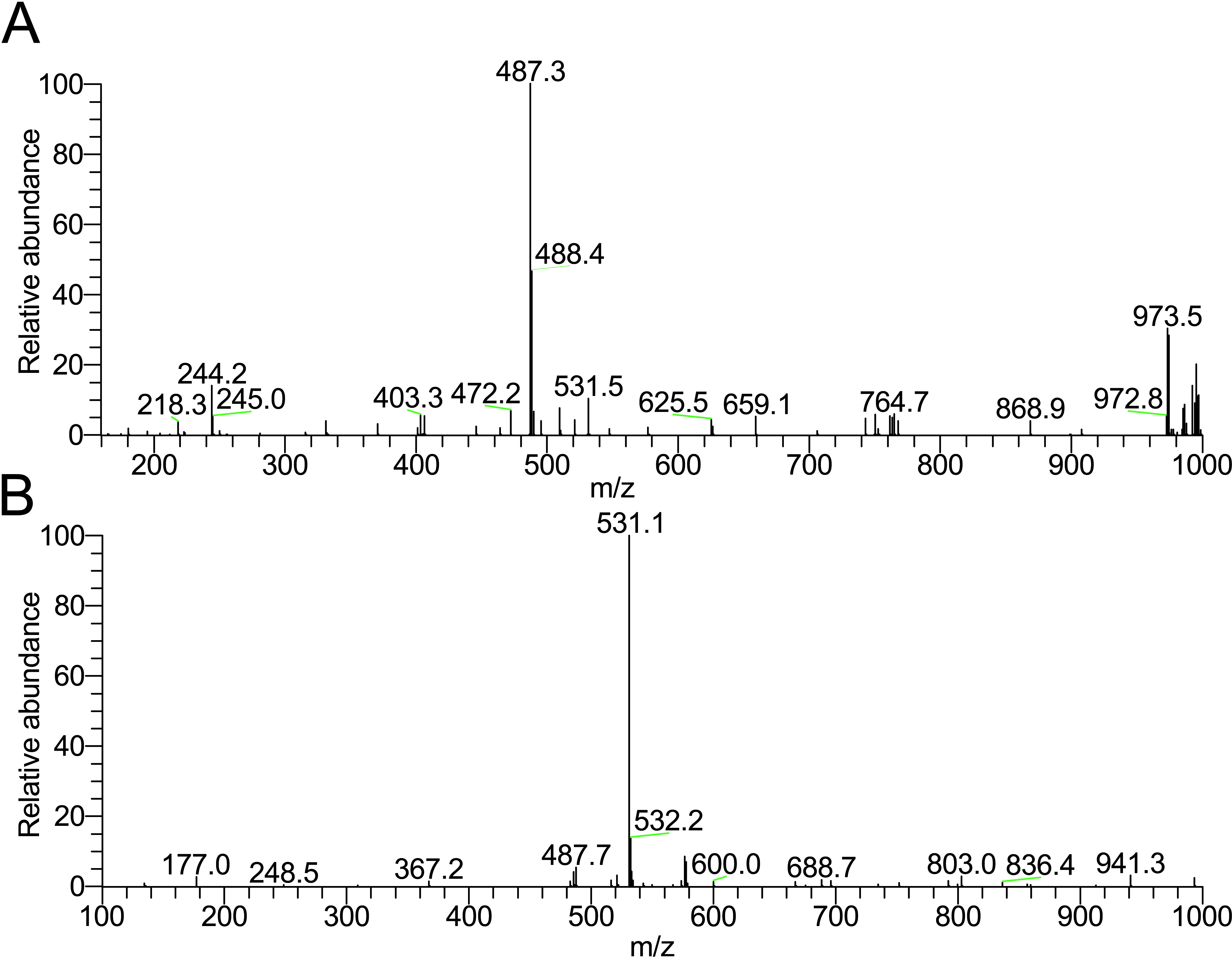

Supplement: S3 Fig — (A) Results for the pseudo molecular ion obtained by positive ionization with a proton; 487.3 Da. (B) Results with negative ionization with formate; 531.1 Da. The calculated mass for the investigated SYPRO Orange ion is 486 Da, in agreement with the structural formula shown in S1 Fig. (TIF) [file pone.0177024.s003.tif]

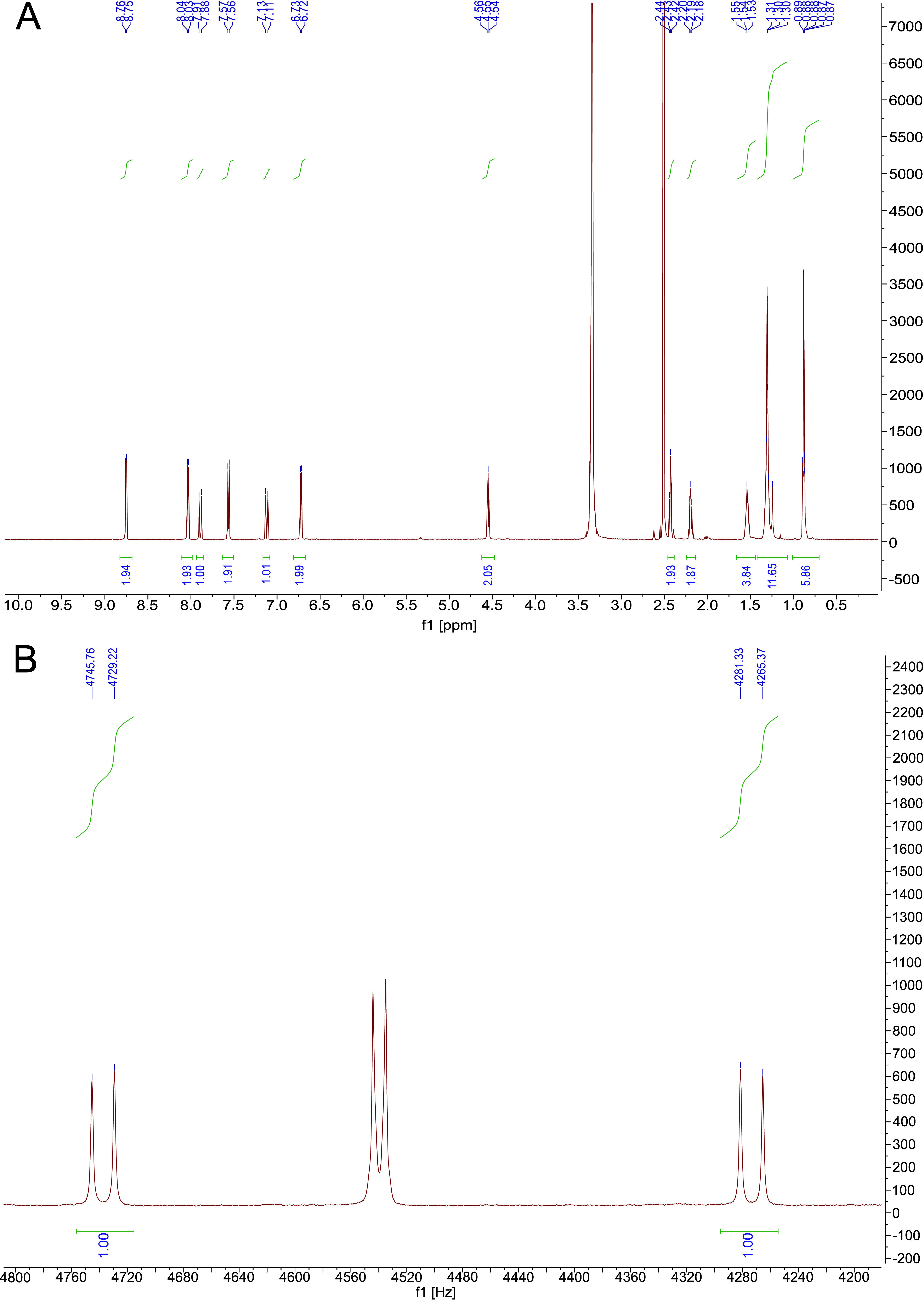

Supplement: S4 Fig — (A) Full proton (1H) NMR spectrum of SYPRO Orange in DMSO-d6. (B) Zoom into the SYPRO Orange-specific part of the spectrum in panel A (between 7.0 and 8.0 ppm); the coupling constant of the protons at the double bond is 3J = 16 Hz, revealing a trans configuration of that bond. (TIF) [file pone.0177024.s004.tif]

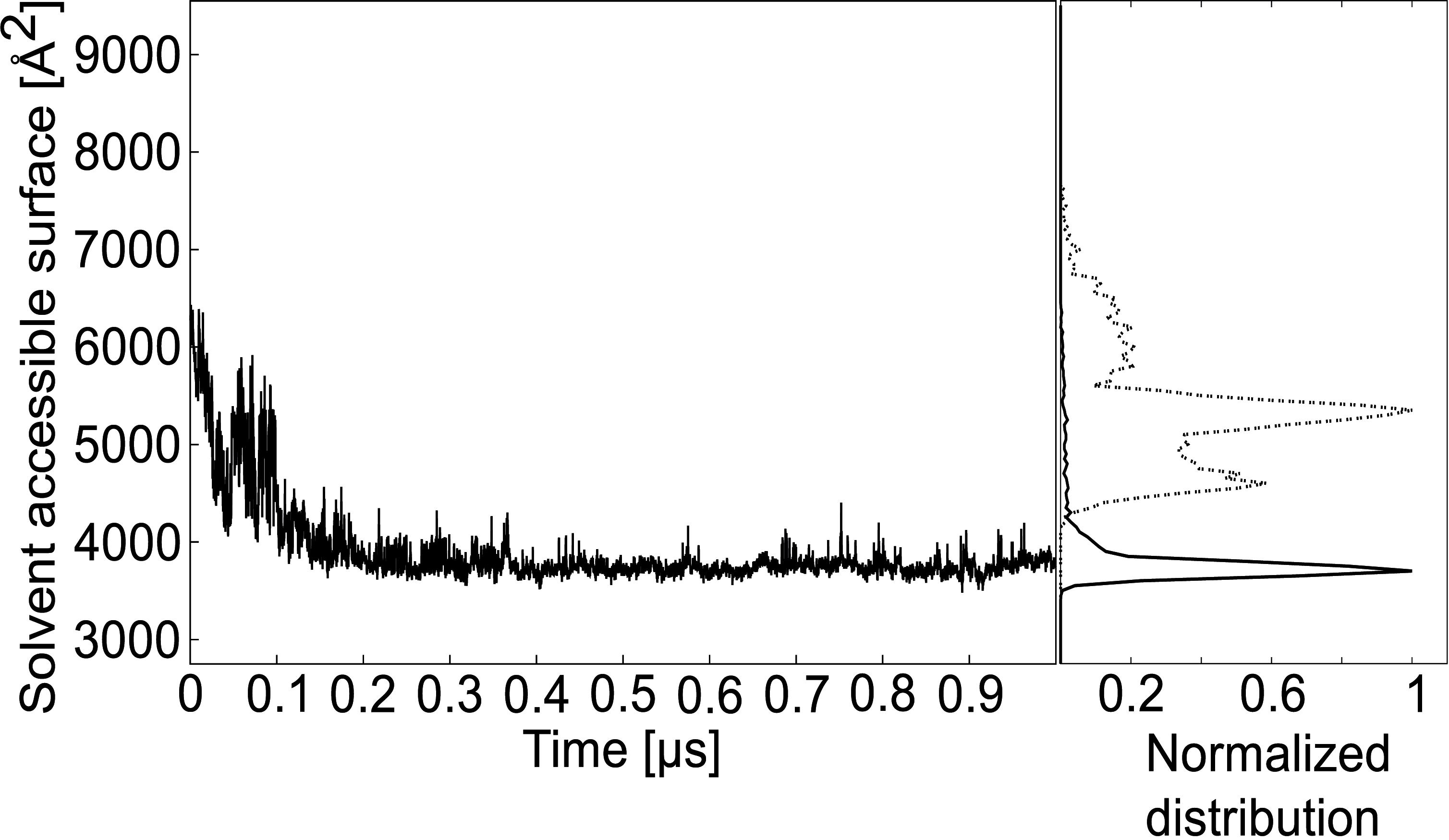

Supplement: S5 Fig — Solvent accessible surface area (SASA) and frequency distribution of the SASA over all 20 EDTA4- molecules in the simulation box (solid line). Here parameters for metal ions from Joung et al. were applied; all other simulation conditions were as in Fig 1. The dashed line in the frequency distribution has been added for comparison (see Fig 3A) and shows the results obtained with the parameters for metal ions from the ions94 library of Amber12. (TIF) [file pone.0177024.s005.tif]

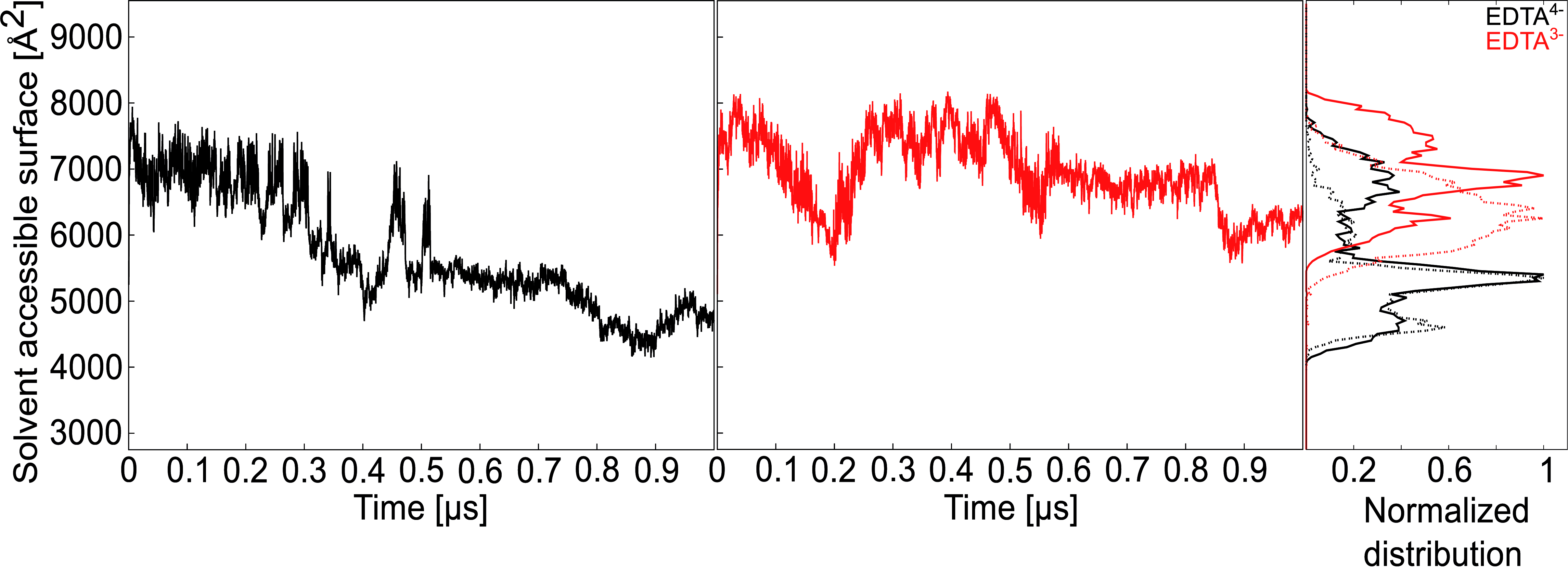

Supplement: S6 Fig — SASA and frequency distribution of the SASA over all 16 EDTA molecules in the simulation box; EDTA4- (black), EDTA3- (red). The dashed line in the frequency distribution has been added for comparison (see Fig 3A) and shows the results obtained with the originally applied concentration. (TIF) [file pone.0177024.s006.tif]

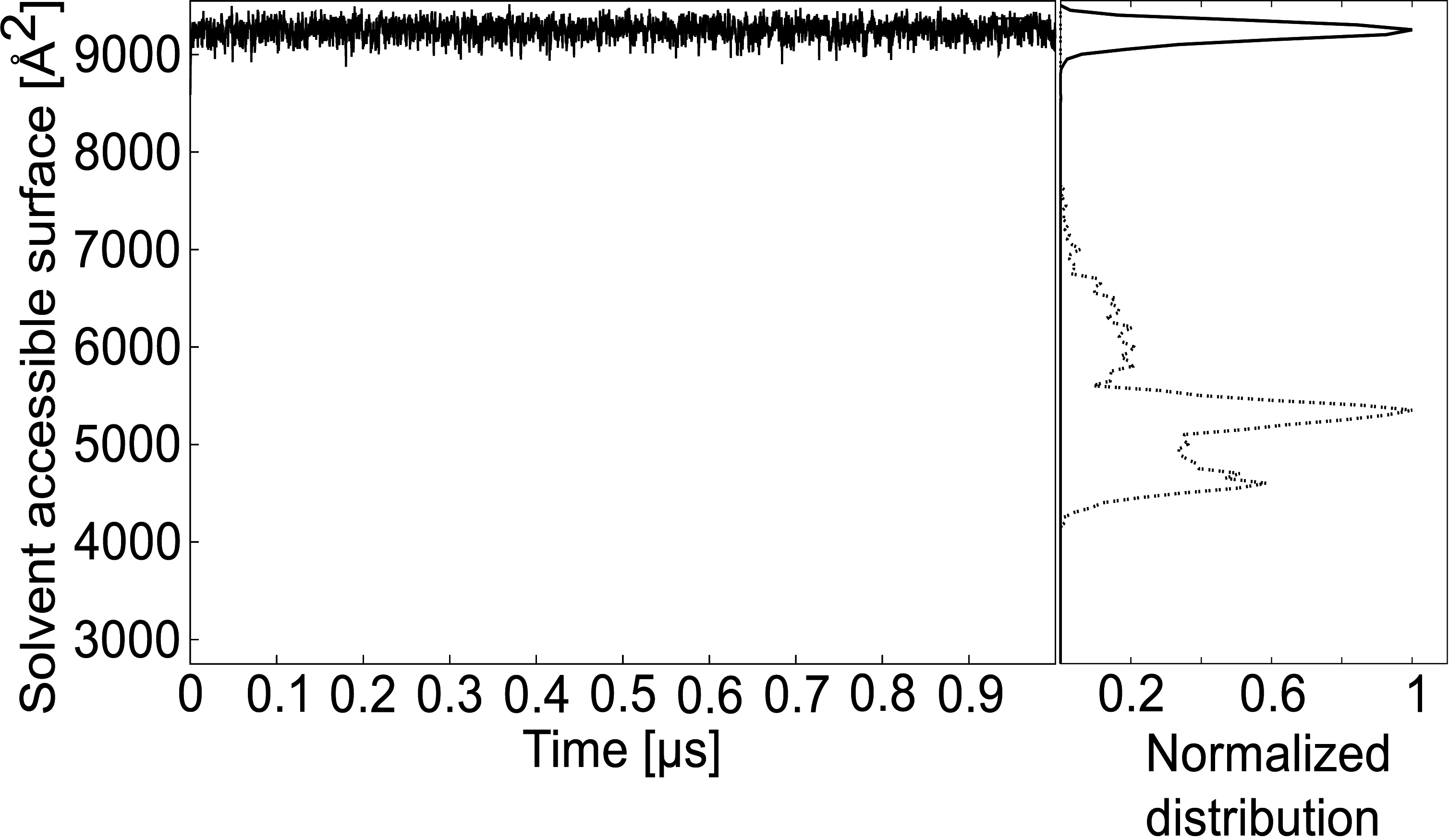

Supplement: S7 Fig — (A) SASA and frequency distribution of the SASA over all 20 EDTA4- molecules in the simulation box in the absence of Na+ counter ions. The dashed lines in the frequency distribution has been added for comparison (see Fig 3A) and shows the results obtained in the presence of the counter ions. (TIF) [file pone.0177024.s007.tif]

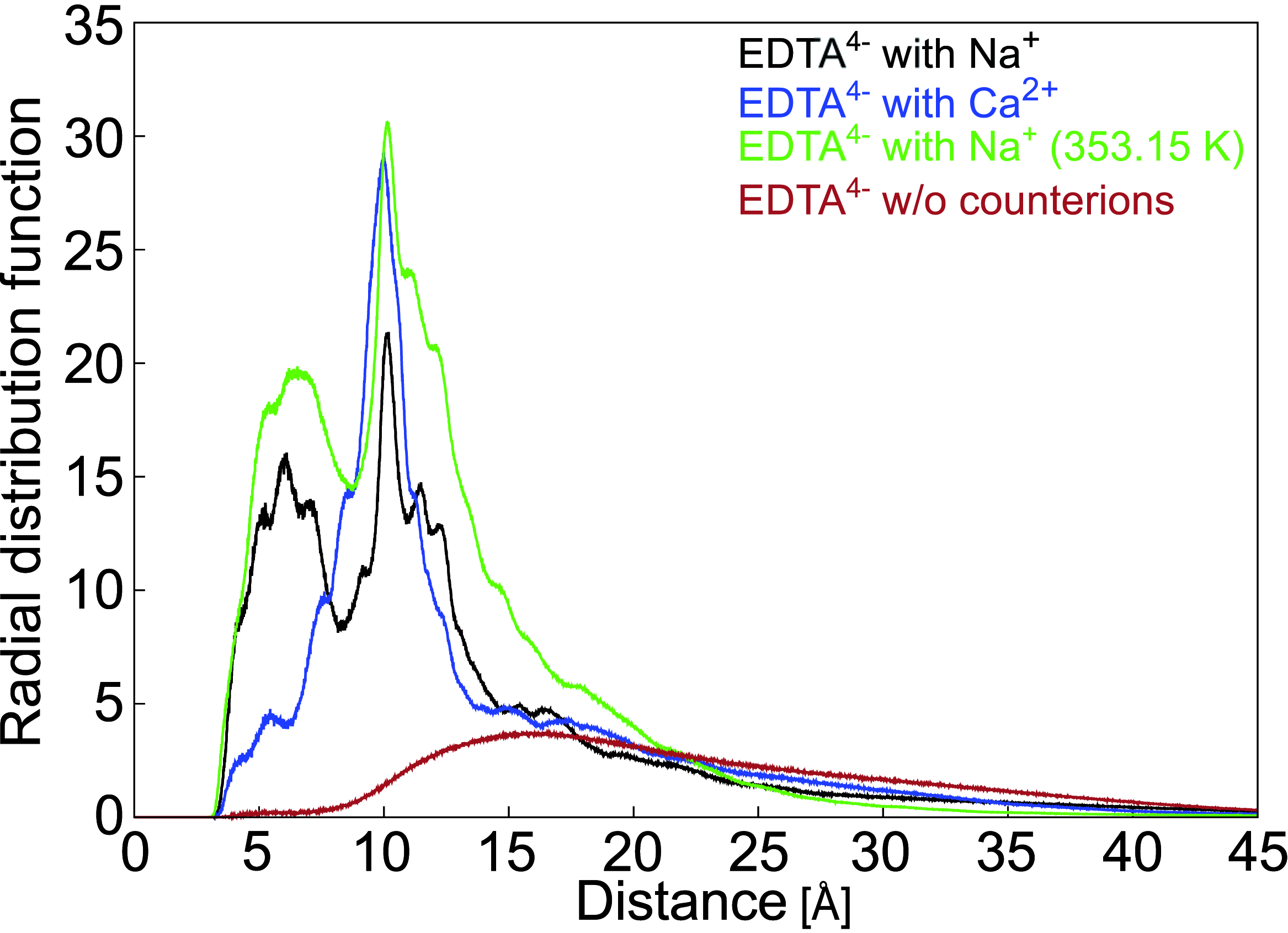

Supplement: S10 Fig — Radial distribution function of carbon atoms of the EDTA4- ethylene moiety, ignoring intramolecular distances, at 300 K in the presence of Na+ (black), in the presence of Ca2+ and Na+ (blue),or in the absence of counterions (brown), and at 353.15 K in the presence of Na+ (green). (TIF) [file pone.0177024.s010.tif]

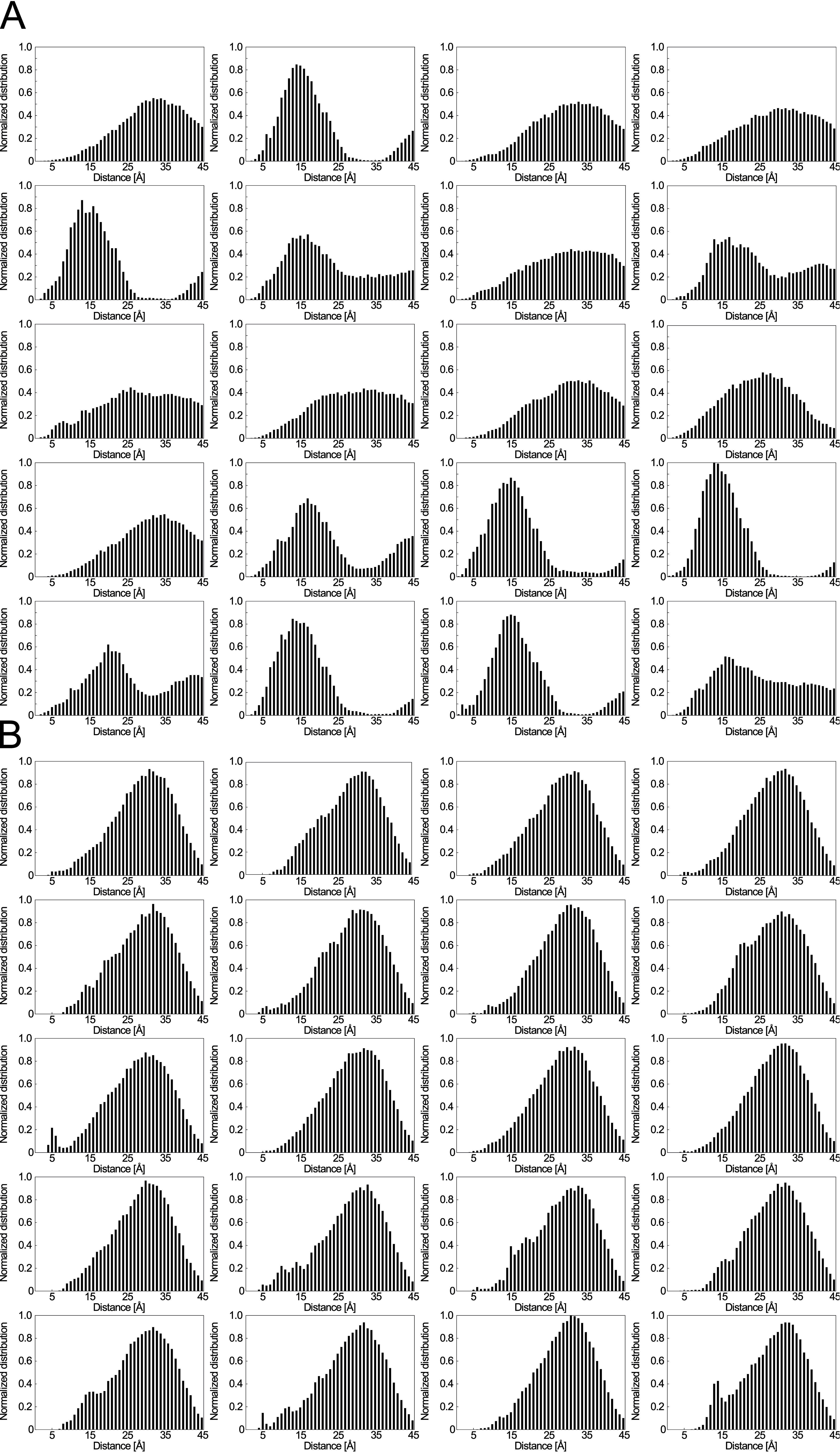

Supplement: S11 Fig — Frequency distributions of distances between the center of ethylene groups of each EDTA4- (A) or EDTA3- (B) molecule and the core region (see S1 Fig) of the nearest SYPRO Orange molecule. (TIF) [file pone.0177024.s011.tif]
