# Supplementary material for: EDTA aggregates induce SYPRO orange-based fluorescence in thermal shift assay
Source: PLoS One. 2017 May 4;12(5):e0177024. doi: 10.1371/journal.pone.0177024 (PMC5417642; doi:10.1371/journal.pone.0177024)
Supplement: S1 Table — (PDF) [file pone.0177024.s012.pdf]

S1 Table. Composition of samples for Thermofluor assay [a] [b].

EDTA[c]	EGTA[c]	Ca^{2+}[c]	pH	Figure
0	0	0	10	2A
1	0	0	10	
10	0	0	10	
20	0	0	10	
30	0	0	10	
40	0	0	10	
50	0	0	10	
60	0	0	10	
70	0	0	10	
80	0	0	10	
90	0	0	10	
100	0	0	10	
100	0	0	7	2B
100	0	0	7.5	
100	0	0	8	
100	0	0	8.5	
100	0	0	9	
100	0	0	9.5	
100	0	0	10	
100	0	0	10.5	
100	0	0	11	
100	0	0	11.5	
100	0	0	12	
100	0	10	10	2C
100	0	50	10	
100	0	100	10	
100	0	200	10	
100	0	500	10	
0	0	10	10	
0	0	50	10	

0	0	100	10	
0	0	500	10	
0	0	0	10	2D
0	30	0	10	
0	60	0	10	
0	60	100	10	

[a] Technical details of Thermofluor assay are described in Materials and Methods.
[b] Components were solved in H_2O.
[c] Concentration, in mM.
