# Supplementary material for: EDTA aggregates induce SYPRO orange-based fluorescence in thermal shift assay
Source: PLoS One. 2017 May 4;12(5):e0177024. doi: 10.1371/journal.pone.0177024 (PMC5417642; doi:10.1371/journal.pone.0177024)
Supplement: S1 Text — (PDF) [file pone.0177024.s013.pdf]

Supporting Methods

Sedimentation velocity analysis

Sedimentation velocity (SV) analysis was carried out to determine the size distributions of EDTA aggregates in the presence of sodium ions. All experiments were performed using a Beckman Optima XL-A ultracentrifuge (Beckman-Coulter, Brea, CA, USA), equipped with a fluorescence detection system (Aviv, Lakewood, NJ, USA) and a four-hole rotor. The detection system is capable of exciting fluorophores at 488 nm and recording the emission fluorescence within 505 and 565 nm. Sample solution which contained 40 mM EDTA, 136 mM Na^+, and 0.4 % SYPRO Orange was prepared freshly, and the pH value of the solution was maintained at 11. For SV analysis, 100 µl samples were loaded into 3 mm titanium double sector cells with quartz glass windows. All cells were then centrifuged at 60,000 rpm at 20 °C for 20 h and radially resolved concentration profiles were collected every 1.5 min. Data were evaluated using the software package Sedfit (Version 15.01b) [1]. In detail, the continuous distribution c(s) Lamm equation model was applied to evaluate size distributions of EDTA aggregates formed in the presence of Na^+. Fitting parameters including buffer density (1.01100 g/cm^3) and viscosity (0.01055 Poise) were calculated using Sednterp (Version 20130813 BETA, http://bitcwiki.sr.unh.edu/index.php/Downloads) [2]. The partial specific volumes of EDTA-Na_4 ($\bar{v}$= 0.5090 cm^3/g) and SYPRO Orange ($\bar{v}$=0.8168 cm^3/g) were calculated according to the method of Durchschlag and Zipper [3], and we used an average partial specific volume (1:5 stoichiometry for SYPRO Orange:EDTA) of 0.5486 cm^3/g for the fitting. The graphical outputs were generated by GUSSI (Version 1.2.1) (http://biophysics.swmed.edu/MBR/software.html) [4], and the final *s*-values were corrected to *s*-values in water at 20 °C ($s_{20,W}$ -values).

LC-Mass spectrometry

SYPRO Orange solution (Analytik Jena AG, Germany) was chromatographically separated with an Agilent 1100 series Liquid Chromatography system (Agilent Technologies, USA) equipped with a quadrupole ion trap LCQ Deca mass spectrometer (ThermoFisher, USA). The mobile phase consisted of water (A) and methanol (B). The elution conditions were: 0-5 min, 10% B; 30 min, 10-100% B; 10 min, 100% B; 15 min, 10% B. The SYPRO Orange molecules were positively ionized and negatively ionized by protons and formate, respectively. The data were analyzed with the software Xcalibur V6.0 (ThermoFisher Scientific, USA), and the results are given as mass-to-charge ratio (m/z) from 0 to 1000.

NMR spectroscopy

SYPRO Orange solution (500 µL, Analytik Jena AG, Germany) was lyophilized and the residue was subsequently dissolved in DMSO-d_6 (0.8 mL). The proton (^{1}H) NMR spectrum was recorded on a Bruker Avance III – 600 (600.22 MHz for ^{1}H). Chemical shifts are given in parts per million (ppm, δ relative to residual solvent peak) or in Hertz (Hz).

References

1. Schuck P. Size-distribution analysis of macromolecules by sedimentation velocity ultracentrifugation and lamm equation modeling. Biophys J. 2000;78(3):1606-19. Epub 2000/02/29. doi: 10.1016/S0006-3495(00)76713-0. PubMed PMID: 10692345; PubMed Central PMCID: PMC1300758.

2. Hurton T, Wright A, Deubler G, Bashir B, Hayes DB, Laue TM, et al. SEDNTERP http://bitcwiki.sr.unh.edu/index.php/Main_Page 2016 [cited 2016 25/11/2016]. Available from: http://bitcwiki.sr.unh.edu/index.php/Main_Page.

3. Durchschlag H, Zipper P. Calculation of the partial volume of organic compounds and polymers. In: Lechner MD, editor. Ultracentrifugation. Darmstadt: Steinkopff; 1994. p. 20-39.

4. Brautigam CA. Calculations and Publication-Quality Illustrations for Analytical Ultracentrifugation Data. Methods in enzymology. 2015;562:109-33. doi: 10.1016/bs.mie.2015.05.001. PubMed PMID: 26412649.
